# Association between dietary fiber intake and chronic kidney disease in adults with and without hypertension in the United States: a cross-sectional study of NHANES 2009–2020

**DOI:** 10.1080/0886022X.2024.2415514

**Published:** 2024-10-16

**Authors:** Chao Zhang, Weimin Yan, Xun Sun, Fansen Lin

**Affiliations:** a Department of Nephrology, Bethune International Peace Hospital, Shijiazhuang, China; b Department of Intensive Care Unit, Bethune International Peace Hospital, Shijiazhuang, China; c Department of Respiratory and Critical Care Medicine, Bethune International Peace Hospital, Shijiazhuang, China; d Department of Patient Management, Bethune International Peace Hospital, Shijiazhuang, China

**Keywords:** Chronic kidney disease, hypertension, dietary fiber intake, cross-sectional study

## Abstract

While previous research has highlighted the potential advantages of increasing dietary fiber intake (DFI) for managing hypertension and chronic kidney disease (CKD), there is a gap in large-scale empirical studies examining the relationship between DFI and CKD among hypertensive and nonhypertensive cohorts independently. This study involved 22,871 participants sourced from the NHANES database spanning 2009 to 2020, who were divided into hypertensive (*n* = 9,861) and nonhypertensive (*n* = 13,010) groups. The analysis revealed a significant inverse correlation between DFI and CKD prevalence across the sample after adjusting for various covariates (OR = 0.98, 95% CI: 0.97–0.99, *p* = 0.001). Within the subset of hypertensive individuals, this inverse association mirrors the findings of the overall sample, indicating that a higher DFI was associated with a reduced occurrence of CKD (OR = 0.97, 95% CI: 0.96–0.99, *p* < 0.001). However, this correlation was not detected in the nonhypertensive group (OR = 0.99, 95% CI: 0.98–1.01, *p* = 0.285). The RCS analysis further confirmed a pronounced nonlinear inverse relationship between DFI and CKD prevalence in both the entire cohort and the hypertensive group but not in the nonhypertensive group. Further scrutiny of the hypertensive group revealed that individuals with a higher DFI had 33% lower odds of CKD progression for the moderate risk level and 36% lower odds for the high to very high risk level. Subgroup analyses confirmed the consistency of these relationships across various demographics. In summary, this investigation revealed a significant inverse relationship between DFI and CKD prevalence in US adults with hypertension, a relationship not observed in nonhypertensive individuals.

## 1. Introduction

Chronic kidney disease (CKD) is a chronic condition that involves functional and/or structural abnormalities of the kidneys that last for more than 3 months. It is characterized by its irreversibility and slow progressive evolution [1]. Epidemiological surveys have revealed that CKD affects more than 10% of the global population, totaling more than 800 million people [2]. CKD seriously harms health-related quality of life and substantially increases the risk of undesirable outcomes, including stroke, myocardial infarction (MI), and heart failure (HF) [3]. CKD has emerged as a critical public health concern. Currently, more than 1.4 million individuals worldwide are receiving renal replacement therapy (RRT) [4]. In some countries, the cost of RRT constitutes 2%-3% of the yearly health care expenditure, even though it is accessed by less than 0.03% of the overall population [5]. However, there is a significant disparity in the global capacity to treat CKD, with only a fraction, often half or less, of the individuals requiring RRT having access to it [6]. Therefore, it is imperative to actively identify preventive measures aimed at reducing the occurrence of CKD.

Hypertension is widely acknowledged as a significant noncommunicable health condition. Its occurrence is increasing globally due to population aging and the increase in lifestyle-related risk factors, such as poor diet. Recent estimates indicate that approximately one-third of adults globally are affected by hypertension (31.1%, 1.39 billion) [7]. Hypertension has long been recognized as a significant causative factor for CKD, contributing to 27% of all cases of end-stage renal disease (ESRD) in the US [8]. Appropriate blood pressure (BP) control has been shown to reduce the likelihood of hypertension leading to CKD, but little is known about other factors that influence the prevalence of CKD in hypertensive patients [9]. Identifying additional causative factors for CKD progression in individuals with hypertension could aid in the development of targeted therapies to delay or inhibit the decline in kidney function.

Dietary fiber refers to the indigestible part of plant material in the diet [10]. It provides various health benefits, including increasing stool bulk, increasing transit time in the intestines, promoting the production of beneficial short-chain fatty acids, and stimulating the growth of beneficial gut bacteria [11]. Research has shown that increased dietary fiber intake (DFI) can lower BP and decrease the likelihood of cardiovascular disease (CVD) [12]. Moreover, dietary fiber plays a vital role in decreasing the production of harmful uremic toxins, protecting the kidneys, and slowing the progression of CKD by influencing various metabolic, immune, and inflammatory processes [13].

Although several previous studies have indicated that a higher DFI may be advantageous for managing hypertension and CKD [14–17], no large-scale study to date has specifically explored the correlation between DFI and CKD in hypertensive and nonhypertensive populations separately. Thus, we utilized data from the National Health and Nutrition Examination Survey (NHANES) from 2009 to 2020 to conduct an extensive cross-sectional survey with the objective of exploring the association between DFI and the occurrence of CKD among Americans with and without hypertension.

## 2. Materials and methods

### 2.1. Data and sample sources

Our study analyzed data from the NHANES, which is a nationwide, typical cross-sectional survey designed to assess the health and nutritional status of adults and children in the United States. The survey employed a stratified, multilevel probabilistic method to gather information. To evaluate the nutritional and physical health status of the participants, standardized in-home interviews, physical examinations, and laboratory tests were performed at mobile examination centers (MECs). The NHANES procedures were approved by the ethics committee of the National Center for Health Statistics (NCHS), with all participants providing signed consent forms.

**Figure 1.**
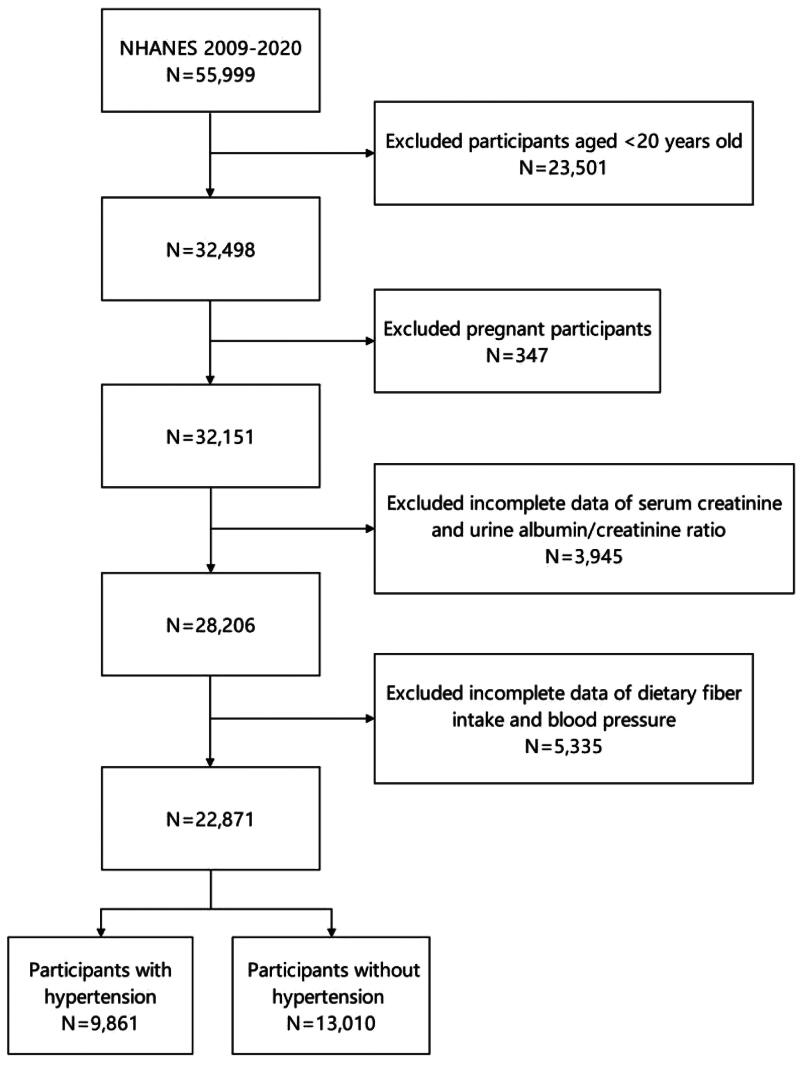
Flow chart of the inclusion and exclusion criteria for the study participants.

From January 2009 to March 2020 (until the start of the COVID-19 pandemic), 55,999 individuals were enrolled in 11.2 NHANES cycles [18]. Participants assigned to the morning MEC underwent blood tests. Among the participants assigned to the morning, those over the age of 12 were asked to fast for at least 8.5 h. The phlebotomist determined the fasting status of each participant through a questionnaire. We eliminated the following participants: (1) those aged less than 20 years (*n* = 23,501), (2) those who were pregnant (*n* = 347), (3) those with missing data on serum creatinine or the urine albumin-to-creatinine ratio (UACR) (*n* = 3,945), and (4) those with missing data on DFI and BP (*n* = 5,335). Ultimately, the study included 22,871 individuals, comprising 9,861 individuals with hypertension and 13,010 individuals without hypertension (Figure 1).

### 2.2. Independent variables

DFI (g/day) was determined through two 24-h dietary recalls. The initial dietary survey, which lasted 24 h, was carried out by qualified data collectors at the MEC, with a follow-up survey conducted over the phone within 3 to 10 days. The mean of these two recall datasets was employed as the DFI for each participant. Participants who lacked dietary fiber information or had data for only one day were excluded. DFI calculations were based on the US Department of Agriculture Food Surveys Nutrient Database, encompassing all food and supplemental sources. Detailed information regarding the dietary evaluation procedures can be found in the NHANES dietary interviewer procedures manuals [19,20].

### 2.3. Grouping and the dependent variable

Systolic blood pressure (SBP) and diastolic blood pressure (DBP) data were collected by averaging three accurate measurements in the MEC. Hypertension was defined as having a mean SBP ≥ 140 mmHg and/or DBP ≥ 90 mmHg, being diagnosed by a physician, or currently taking medication for hypertension [21]. The estimated glomerular filtration rate (eGFR) was measured *via* the 2021 CKD Epidemiology Collaboration creatinine equation [22]. CKD was defined as an eGFR < 60 mL/min/1.73 m^2^ or a UACR ≥ 30 mg/g [23]. The assessment of CKD progression risk was based on the eGFR and albuminuria categories, which were categorized as low, moderate, high, or very high risk [24].

### 2.4. Covariates

The following covariates that may influence the association between DFI and CKD were included. The demographic characteristics included sex, age, race, education, marital status, and the ratio of family income to poverty (PIR). A fasting blood glucose (FBG) level of ≥ 125 mg/dL, self-disclosed diabetes, or current use of a hypoglycemic agent were considered indicators of diabetes mellitus (DM) [25]. The smoking status was categorized as follows: nonsmokers (< 100 cigarettes throughout their lifetime), ever smokers (≥ 100 cigarettes throughout their lifetime, not currently smoking), and current smokers (≥ 100 cigarettes throughout their lifetime, sometimes or daily smoking) [26]. Alcohol consumption was divided into three categories: light, moderate and heavy [27]. BMI was categorized on the basis of the WHO’s standard as < 25, 25–29.9, and ≥ 30 kg/m^2^, corresponding to a healthy weight, excess weight, and obesity, respectively [28]. Sedentary time was categorized as < 3, 3–5.9, or ≥ 6 h/day, with data > 18 h/day excluded. Serum cotinine, alanine aminotransferase (ALT), serum albumin (ALB), total cholesterol (TC), and triglyceride (TG) levels were also considered factors. Hypercholesterolemia was defined as a total cholesterol level ≥ 240 mg/dl [29]. Data on cardiac disease and cancer were obtained from questionnaires. Cardiac disease was defined as having HF, coronary heart disease (CHD), or MI. The study also included the systemic immune-inflammation index (SII), a novel marker of systemic inflammation that is favorably correlated with CKD [30].

### 2.5. Statistical analyses

We used the WTMEC2YR (2009–2016) and WTMECPRP (2017–2020) variables, as recommended by the NHANES, to provide weighting for all the data in our intricate sampling investigation. To assess variances in fundamental traits among the various CKD and DFI groups, we applied the weighted t test for continuous variables and the weighted chi-square test for categorical variables. Continuous variables are reported as the means ± standard deviations, and categorical variables are shown as tallies (percentages). Weighted generalized linear regression was used to assess the association between DFI and CKD with different models. Model 1 did not include adjustments for any covariates; Model 2 was adjusted for sex, age, race, education, marriage status and PIR; Model 3 was additionally adjusted for smoking behavior, alcohol consumption, BMI, caloric intake, sedentary time, serum cotinine, ALT, ALB, TC, TG, SII, DM, cardiac disease, and cancer, expanding on the adjustments in Model 2. The outcomes are reported as ORs and 95% CIs. The relationship between DFI and CKD was assessed *via* RCS with six knots to examine the dose–response pattern. Interaction analyses were conducted to explore the variations in connections among different subgroups. All the simulations and analyses were conducted *via* R software (version 4.3.2). A *p* value of less than 0.05 was considered statistically significant.

## 3. Results

### 3.1. Participants’ fundamental attributes

The study included a large sample of 22,871 individuals, which can be indicative of a population size of approximately 1.78 × 10^8^ on the basis of weighting calculations. On average, the participants were 48.43 ± 16.82 years old, of which 48.11% were men and 51.89% were women. The mean DFI was 17.25 ± 9.27 g/day, and 11.60% of the participants were classified as having CKD. The clinical features of the individuals were stratified by CKD status (Table 1). Individuals with CKD differed greatly from those without CKD in terms of age, race, education, marital status, PIR, smoking behavior, alcohol consumption, BMI, caloric intake, DM, hypertension, sedentary time, cardiac disease, cancer, and DFI (*p* < 0.05). Sex and hypercholesterolemia were not significantly different between the two groups. Compared with those without CKD, individuals with CKD were older, non-Hispanic Black, had a high school/middle school education or lower, were widowed/divorced/separated, had a lower PIR, had a lower dietary energy intake, were ever smokers, had mild alcohol consumption, had a BMI ≥ 30, had sedentary time ≥ 6 h/day, and had DM, hypertension, cardiac disease, or cancer, as well as a lower DFI.

**Table 1.** Fundamental attributes of weighted samples from the CKD and non-CKD groups.

	Overall N = 22871	CKD N = 3454	Non-CKD N = 19417	*p* Value
Sex (%)				0.314
Male	48.11	49.31	47.95	
Female	51.89	50.69	52.05	
Age (years)	48.43 ± 16.82	59.18 ± 17.33	47.02 ± 16.23	< 0.001
Race (%)				< 0.001
Mexican American	8.02	7.89	8.03	
Non-Hispanic Black	10.56	14.35	10.07	
Non-Hispanic White	67.88	65.52	68.19	
Other Hispanic	5.97	5.17	6.08	
Other races	7.56	7.06	7.62	
Education (%)				< 0.001
College or more	64.00	54.83	65.20	
High school	22.78	25.37	22.44	
Middle school or lower	13.17	19.71	12.31	
Marital status (%)				< 0.001
Never married	14.08	14.02	14.08	
Married or with partner	78.23	69.26	79.51	
Widowed, divorced or separated	7.69	16.71	6.41	
PIR	3.07 ± 1.64	2.72 ± 1.62	3.12 ± 1.64	< 0.001
Smoke status (%)				< 0.001
Never smokers	56.53	50.25	57.35	
Ever smokers	25.79	33.74	24.75	
Current smokers	17.68	16.01	17.90	
Alcohol consumption (%)				< 0.001
Mild	87.02	90.65	86.55	
Moderate	8.40	5.26	8.80	
Heavy	4.58	4.08	4.65	
Dietary energy intake (kcal)	2088.77 ± 807.36	1924.43 ± 769.61	2110.34 ± 809.73	< 0.001
Dietary potassium intake (mg)	2667.88 ± 1085.62	2510.06 ± 986.93	2688.59 ± 1096.25	< 0.001
DM (%)				< 0.001
Yes	12.37	34.46	9.50	
No	87.63	65.54	90.50	
Hypertension (%)				< 0.001
Yes	38.13	66.15	34.46	
No	61.87	33.85	65.54	
BMI (%)				<0.001
< 25	28.11	22.42	28.85	
25–30	37.82	32.6	38.49	
≥ 30	34.07	44.99	32.66	
Sedentary time (%)				0.046
< 3 h/day	11.68	10.3	11.86	
3–5.9 h/day	46.41	45.23	46.57	
≥ 6 h/day	41.91	44.47	41.57	
Hypercholesterolemia (%)				0.286
Yes	11.93	12.71	11.83	
No	88.07	87.29	88.17	
Cardiac disease (%)				< 0.001
Yes	2.37	8.77	1.53	
No	97.63	91.23	98.47	
Cancer (%)				< 0.001
Yes	10.96	17.79	10.07	
No	89.04	82.21	89.93	
DFI (g/day)	17.25 ± 9.27	15.89 ± 8.22	17.43 ± 9.38	< 0.001

Mean ± SD for continuous variables, percentages (%) for categorical variables.

PIR = poverty income ratio, BMI = body mass index, DM = diabetes mellitus, DFI = dietary fiber intake.

Individuals were distinguished based on DFI tertiles (T1 < 12.00 g/day, T2 = 12.00–18.75 g/day, T3 ≥ 18.75 g/day) (Table 2). Significant differences were found among the DFI tertiles in terms of sex, age, race, education, marital status, PIR, smoking status, alcohol consumption, BMI, dietary energy intake, sedentary time, DM, hypertension, cardiac disease, and CKD (*p* < 0.05). Hypercholesterolemia and cancer incidence did not differ among the three groups. The participants in the T1 group (lower DFI) tended to be female, younger, non-Hispanic Black/other Hispanic, never married/widowed/divorced/separated, and current smokers, as well as have a high school/middle school education or lower, a lower PIR, moderate or heavy alcohol consumption, a lower dietary energy intake, a sedentary time < 6 h/day, and DM, hypertension or cardiac disease. Additionally, the prevalence of CKD was greater among individuals in the low DFI tertile than in those in the high DFI tertile (T1, 23.67%; T2, 20.36%; T3, 16.95%, *p* < 0.001).

**Table 2.** Fundamental attributes of the weighted sample by DFI tertiles.

	Overall N = 22871	T1 < 12.00 g/day N = 7525	T2 12.00–18.75 g/day N = 7562	T3 ≥ 18.75 g/day N = 7784	*p* Value
Sex (%)					< 0.001
Male	48.11	39.14	45.75	58.19	
Female	51.89	60.86	54.25	41.81	
Age (years)	48.43 ± 16.82	47.42 ± 17.42	48.92 ± 16.91	48.83 ± 16.16	< 0.001
Race (%)					< 0.001
Mexican American	8.02	5.54	7.29	10.88	
Non-Hispanic Black	10.56	15.51	10.22	6.61	
Non-Hispanic White	67.88	65.53	69.88	67.99	
Other Hispanic	5.97	6.31	5.40	6.25	
Other races	7.56	7.12	7.22	8.27	
Education (%)					< 0.001
College or more	64.00	54.51	64.11	72.11	
High school	22.78	28.87	24.03	16.28	
Middle school or lower	13.17	16.55	11.82	11.54	
Marital status (%)					< 0.001
Never married	14.08	17.90	14.15	11.30	
Married or with partner	78.23	72.09	77.66	83.11	
Widowed, divorced or separated	7.69	10.01	8.20	5.58	
PIR	3.07 ± 1.64	2.69 ± 1.64	3.15 ± 1.61	3.33 ± 1.62	< 0.001
Smoke status (%)					< 0.001
Never smokers	56.53	50.84	57.12	60.89	
Ever smokers	25.79	23.05	26.37	27.61	
Current smokers	17.68	26.11	16.51	11.50	
Alcohol consumption (%)					< 0.001
Mild	87.02	83.96	88.41	88.12	
Moderate	8.40	9.92	7.49	8.05	
Heavy	4.58	6.11	4.11	3.83	
Dietary energy intake (kcal)	2088.77 ± 807.36	1601.99 ± 586.58	2053.85 ± 638.97	2545.33 ± 860.96	< 0.001
DM (%)					0.009
Yes	12.37	13.64	12.15	11.49	
No	87.63	86.36	87.85	88.51	
Hypertension (%)					< 0.001
Yes	38.13	40.59	38.48	35.65	
No	61.87	59.41	61.52	64.35	
BMI (%)					< 0.001
< 25	28.11	26.75	26.5	30.86	
25–30	37.82	37.27	37.65	38.46	
≥ 30	34.07	35.98	35.85	30.68	
Sedentary time (%)					0.019
< 3 h/day	11.68	12.63	10.98	11.54	
3–5.9 h/day	46.41	47.54	46.38	45.47	
≥ 6 h/day	41.91	39.83	42.64	42.99	
Hypercholesterolemia (%)					0.369
Yes	11.93	11.86	12.49	11.45	
No	88.07	88.14	87.51	88.55	
Cardiac disease (%)					< 0.001
Yes	2.37	3.06	2.43	1.71	
No	97.63	96.94	97.57	98.29	
Cancer (%)					0.312
Yes	10.96	10.39	11.49	10.94	
No	89.04	89.61	88.51	89.06	
CKD (%)					< 0.001
Yes	11.60	14.01	11.43	9.68	
No	88.4	85.99	88.57	90.32	

Mean ± SD for continuous variables, percentages (%) for categorical variables.

PIR = poverty income ratio, BMI = body mass index, DM = diabetes mellitus, CKD = chronic kidney disease.

We further divided all participants into two groups: hypertensive (*N* = 9861) and nonhypertensive (*N* = 13010). We then analyzed baseline characteristics using CKD and DFI tertiles as column-stratified variables separately (Tables S1–S4). Among the hypertensive participants, the prevalence of CKD was 20.12%, and the DFI in the CKD group (15.32 ± 7.67 g/day) was lower than that in the non-CKD group (16.89 ± 8.62 g/day) (*p* < 0.001). When the hypertensive participants were divided into 3 groups according to DFI (T1 < 11.55 g/day, T2 = 11.55–18.08 g/day, T3 ≥ 18.08 g/day), the prevalence of CKD was greater in the low-DFI group than in the high-DFI group (T1, 23.67%; T2, 20.36%; T3, 16.95%) (*p* < 0.001). Similarly, among nonhypertensive participants, the prevalence of CKD was 6.35%, and there were no notable differences in DFI between the CKD group (17.00 ± 9.09 g/day) and the non-CKD group (17.72 ± 9.74 g/day) (*p* = 0.097). When the nonhypertensive participants were divided into 3 groups according to DFI (T1 < 12.35 g/day, T2 = 12.35–19.35 g/day, T3 ≥ 19.35 g/day), the prevalence of CKD in the low-DFI group was not significantly different from that in the high-DFI group (T1, 7.22%; T2, 5.84%; T3, 6.06%) (*p* = 0.125).

### 3.2. Association between DFI and CKD

We conducted weighted generalized linear regression analyses of the relationship between DFI and CKD prevalence among all participants, both hypertensive and nonhypertensive individuals (Table 3). The models were applied as follows: Model 1, without covariate adjustment; Model 2, adjusted for age, sex, race, marriage, education, and PIR; and Model 3, adjusted for all covariates. Among all participants, the correlations between DFI and CKD prevalence were statistically significant in Model 1 (OR = 0.98, 95% CI: 0.97–0.99, *p* < 0.001), Model 2 (OR = 0.97, 95% CI: 0.96–0.98, *p* < 0.001), and Model 3 (OR = 0.98, 95% CI: 0.97–0.99, *p* = 0.001). The ORs for T2 and T3 were 0.79 (95% CI: 0.71–0.89) and 0.66 (95% CI95% CI 0.58–0.75) for Model 1, 0.77 (95% CI: 0.65–0.91) and 0.62 (95% CI: 0.51–0.75) for Model 2, and 0.81 (95% CI: 0.67–0.99) and 0.70 (95% CI: 0.57–0.87) for Model 3. The *p* values were < 0.001, < 0.001, and 0.003, respectively. This result indicated a decreasing trend in the prevalence of CKD as DFI increased. In Model 3, individuals in the T3 subgroup presented 30% lower odds of CKD than those in the T1 group.

**Table 3.** Correlations between DFI and CKD prevalence, weighted.

	OR (95% CI)		
All participants	Model 1	Model 2	Model 3
DFI (g/day)	0.98 (0.97, 0.99)	0.97 (0.96, 0.98)	0.98 (0.97, 0.99)
*p* Value	< 0.001	< 0.001	0.001
DFI tertiles			
T1, < 12.00 g/day	Ref	Ref	Ref
T2, 12.00–18.75 g/day	0.79 (0.71, 0.89)	0.77 (0.65, 0.91)	0.81 (0.67, 0.99)
T3, ≥ 18.75 g/day	0.66 (0.58, 0.75)	0.62 (0.51, 0.75)	0.70 (0.57, 0.87)
*p* for trend	< 0.001	< 0.001	0.003
Hypertensive participants			
DFI (g/day)	0.98 (0.97, 0.98)	0.97 (0.96, 0.98)	0.97 (0.96, 0.99)
*p* Value	< 0.001	< 0.001	< 0.001
DFI tertiles			
T1, < 11.55 g/day	Ref	Ref	Ref
T2, 11.55–18.08 g/day	0.82 (0.71, 0.96)	0.78 (0.61, 0.99)	0.83 (0.63, 1.07)
T3, ≥ 18.08 g/day	0.66 (0.58, 0.75)	0.59 (0.48, 0.74)	0.67 (0.52, 0.86)
*p* for trend	< 0.001	< 0.001	0.004
Non-hypertensive participants			
DFI (g/day)	0.99 (0.98, 1.00)	0.99 (0.98, 1.00)	0.99 (0.98, 1.01)
*p* Value	0.120	0.106	0.285
DFI tertiles			
T1, <12.35 g/day	Ref	Ref	Ref
T2, 12.35–19.35 g/day	0.80 (0.63, 1.01)	0.80 (0.56, 1.15)	0.85 (0.57, 1.27)
T3, ≥ 19.35 g/day	0.83 (0.67, 1.03)	0.77 (0.59, 1.02)	0.83 (0.61, 1.12)
*p* for trend	0.100	0.094	0.250

Model 1: adjusted for no covariates; Model 2: adjusted for sex, age, race, education, marriage and PIR; Model 3: adjusted for all covariates. DFI = dietary fiber intake.

In hypertensive participants, we consistently observed a correlation, with DFI showing an inverse association with the prevalence of CKD in Model 1 to Model 3 (*p* < 0.001). In Model 3, individuals in the T3 group had 33% lower odds of CKD than those in the T1 group (*p* for trend = 0.004). However, in nonhypertensive participants, no correlation was demonstrated between DFI and CKD. In Model 1 to Model 3, neither dietary fiber nor its tertiles were significantly associated with the prevalence of CKD (*p* ≥ 0.05).

**Figure 2.**
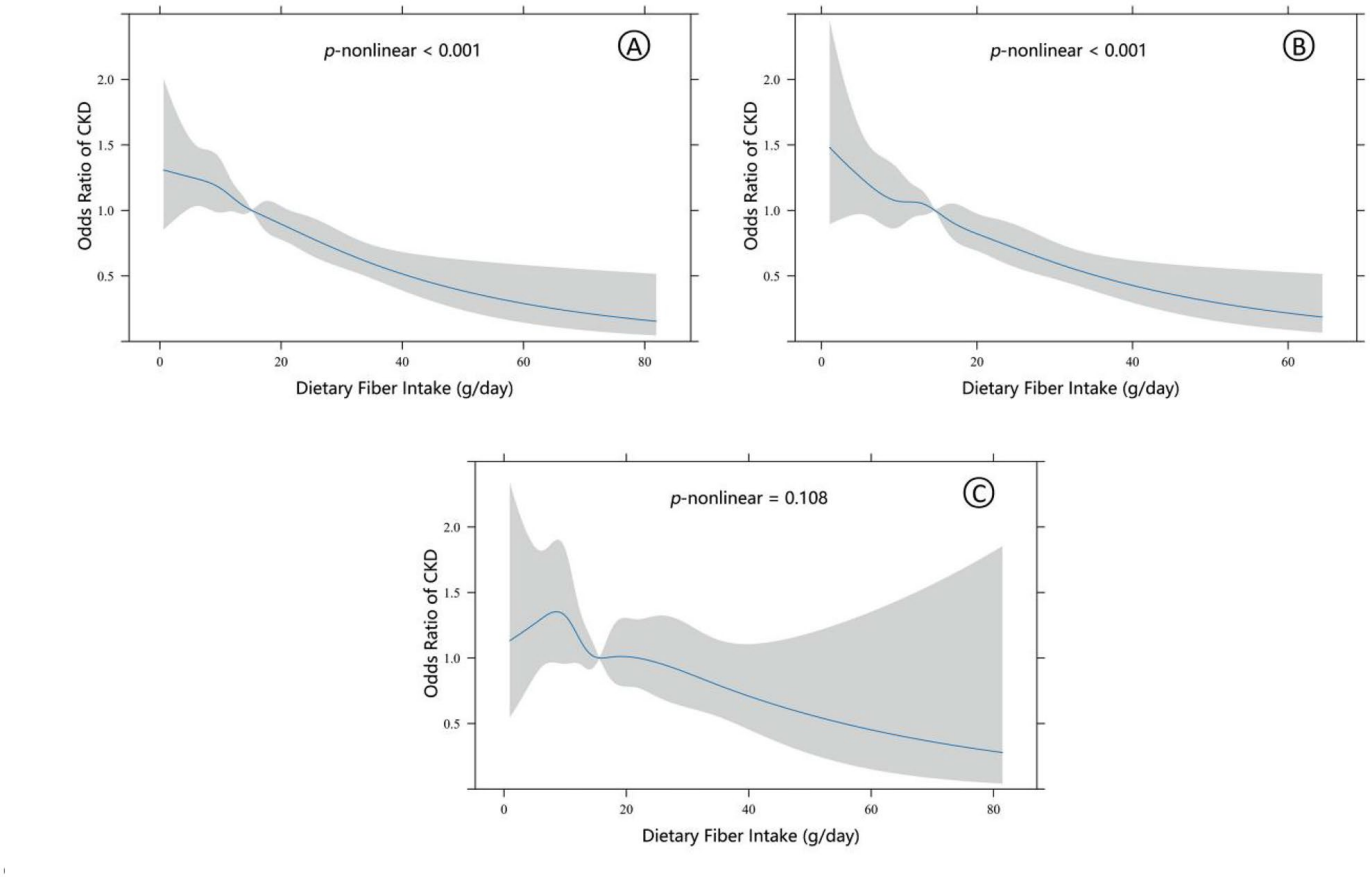
Restricted cubic spline (RCS) plot of the association between DFI and CKD prevalence. (a) In all participants, there was a nonlinear inverse association between DFI and CKD prevalence; (b) in patients with hypertension, there was a nonlinear inverse association between DFI and CKD prevalence; (c) in patients without hypertension, there was no nonlinear inverse association between DFI and CKD prevalence. The adjustment factors are the same as those presented in Model 3. The solid line and shading represent the odds ratio and its 95% confidence intervals, respectively.

Additionally, we analyzed the dose–response relationship between DFI and CKD prevalence *via* RCS and observed a robust nonlinear negative relationship in all participants and hypertensive participants (*p*-nonlinear < 0.001). However, this relationship was not as pronounced in nonhypertensive participants (*p*-nonlinear = 0.108) (Figure 2).

To further clarify the relationship between DFI and CKD, we replaced the dependent variable with the eGFR and UACR and analyzed the correlation between DFI and these variables separately. We separated eGFRs into < 60 and ≥ 60 mL/min/1.73 m^2^. UACR was divided into < 30 and ≥ 30 mg/g. The findings revealed a negative association between DFI and both the eGFR and the UACR among all participants and those with hypertension (*p* value and *p* value for trend < 0.05). However, this correlation was not significant among nonhypertensive individuals (*p* value and *p* for trend > 0.05) (Tables S5 and S6). The trends of the corresponding RCS curves were also consistent with the above results (Figures S1 and S2).

### 3.3. Relationship between DFI and CKD progression risk

Based on the aforementioned findings, we performed a more in-depth analysis on hypertensive individuals, utilizing weighted generalized linear regression to explore the association between the DFI tertiles and the risk of CKD progression (Table 4). Within the hypertensive cohort, the numbers of individuals classified as having a low, moderate, high, or very high risk for CKD progression were 7,396, 1,671, 487, and 307, respectively. Given the limited number of patients falling into the high- and very high-risk categories, these two levels were combined into a single category. The outcomes from Model 3 indicated ORs of 0.67 (95% CI: 0.49–0.92, *p* for trend = 0.019) for T3 in the moderate-risk group and 0.64 (95% CI: 0.43–0.96, *p* for trend = 0.034) for the high- and very high-risk groups (using T1 as the reference). In conclusion, an inverse association was found between a high DFI and the risk of CKD progression among hypertensive individuals. Furthermore, in the multivariable adjusted model, a high DFI (T3) was associated with a 33% reduction in odds for moderate risk and a 36% reduction in odds for high and very high risk compared with a low DFI (T1).

**Table 4.** Relationships between the DFI tertiles and CKD progression risk, weighted.

	OR (95% CI)			
Stratification factors	T1 < 11.55 g/day N = 3240	T2 11.55 ∼ 18.08 g/day N = 3268	T3 ≥ 18.08 g/day N = 3353	*p* for trend
CKD cases with low risk of progression(*N* = 7396)	Ref	Ref	Ref	
CKD cases with moderate risk of progression(*N* = 1671)				
Model 1	Ref	0.87 (0.72, 1.05)	0.71 (0.61, 0.83)	< 0.001
Model 2	Ref	0.77 (0.57, 1.05)	0.61 (0.46, 0.81)	0.001
Model 3	Ref	0.82 (0.59, 1.14)	0.67 (0.49, 0.92)	0.019
CKD cases with high and very high risk of progression(*N* = 794)				
Model 1	Ref	0.73 (0.61, 0.87)	0.54 (0.43, 0.68)	< 0.001
Model 2	Ref	0.82 (0.63, 1.07)	0.55 (0.40, 0.76)	< 0.001
Model 3	Ref	0.90 (0.63, 1.27)	0.64 (0.43, 0.96)	0.034

Refer to Table 3 for details.

### 3.4. Subgroup analysis

**Figure 3.**
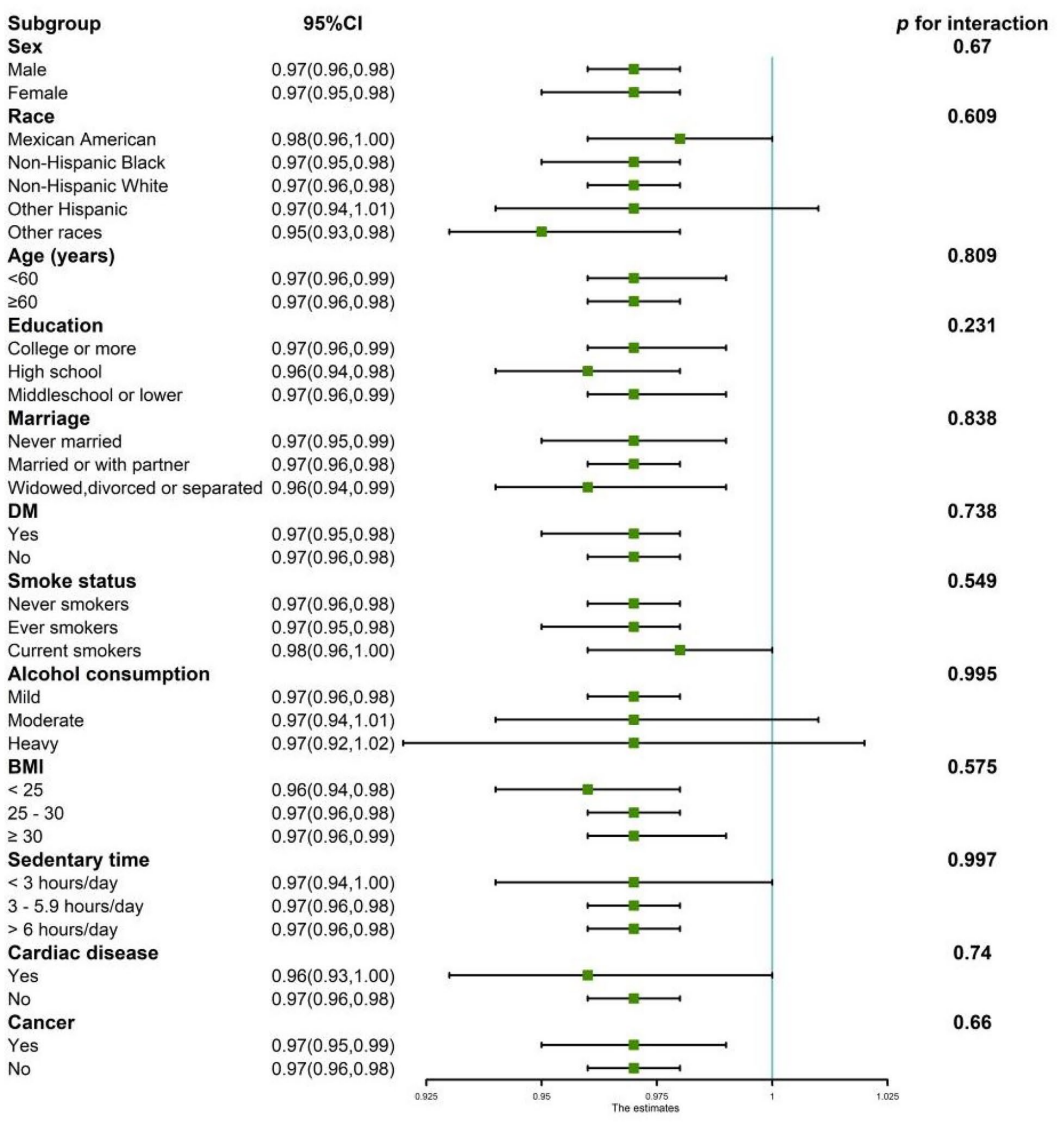
Subgroup analysis of the relationship between DFI and CKD prevalence. The adjustment factors are the same as those presented in Model 3, except for the factor defining the subgroup. The figure shows that factors such as age, sex, race, education, marital status, smoking status, and alcohol consumption do not influence the negative correlation between DFI and CKD prevalence. BMI, body mass index; DM, diabetes mellitus.

We conducted subgroup analyses and presented the results *via* forest plots (Figure 3). The subgroups were categorized by sex, age, race, education, marital status, smoking status, alcohol consumption, BMI, sedentary time, DM, cardiac disease, and cancer. The findings revealed a consistent negative correlation between DFI and the prevalence of CKD across all subgroups (*p* > 0.05 for all interactions).

## 4. Discussion

In this research, we intended to examine the association between DFI and the prevalence of CKD in the adult population of the US. The study included 22,871 individuals, with an average DFI of 17.25 ± 9.27 g/day and an overall prevalence of CKD of 11.6%. Using weighted generalized linear regression and adjusting for relevant covariates, a higher DFI was found to be independently associated with a lower prevalence of CKD. However, when we divided all the participants into two groups according to whether they had hypertension, we found that the negative correlation between DFI and the prevalence of CKD was present only in the hypertensive group, whereas this negative correlation disappeared in the nonhypertensive group. These findings suggest that adding dietary fiber to the diet could lower the occurrence of CKD among hypertensive patients. Interestingly, potassium intake was lower in participants with CKD than in those without CKD. A plausible explanation for this result is that, to prevent the development of hyperkalemia, people with CKD are usually restricted from potassium-rich foods, such as vegetables and fruits, which are also typically rich in dietary fiber. Thus, the inverse correlation between DFI and CKD prevalence may be due to medical reasons.

The current findings from studies examining the relationship between dietary fiber and CKD are inconclusive. A prospective study conducted in Tehran revealed that increased total fiber intake, especially from beans and vegetables, was linked to a reduced risk of developing CKD [14]. Additionally, a study involving 1,110 individuals aged 70–71 years revealed that increased total fiber consumption was positively correlated with the eGFR and was linked to a decreased risk of death in individuals with kidney dysfunction [16]. However, data from the Blue Mountains Eye Study, which involved 2,600 individuals aged ≥ 50 years, revealed that after adjusting for factors such as BMI, smoking, and alcohol consumption, no substantial correlation was found between DFI and the incidence of moderate CKD [15]. Importantly, the aforementioned studies solely examined the participants’ eGFRs and did not concurrently investigate the correlation between DFI and urinary protein or CKD progression risk stratification. Additionally, they did not differentiate between hypertensive and nonhypertensive participants.

Therefore, we conducted a cross-sectional study using the NHANES database to investigate the correlation between DFI and the prevalence of CKD in US adults aged ≥ 20 years. CKD was defined as an eGFR < 60 mL/min/1.73 m^2^ and a UACR ≥ 30 mg/g [31]. Furthermore, we analyzed the relationship between DFI and eGFR, as well as the relationship between DFI and UACR, separately. The results showed that DFI was negatively correlated with the prevalence of CKD, the eGFR, and the UACR. However, this negative correlation was present only in hypertensive participants and not in nonhypertensive participants. Further analysis of the hypertensive participants revealed that, compared with a low DFI, a high DFI was associated with decreased odds of CKD progression in individuals at moderate, high and very high risk. In subgroup analyses of hypertensive participants, the inverse relationship between DFI and the prevalence of CKD remained consistent across populations with diverse demographic characteristics, lifestyle choices, and medical conditions.

The mechanisms underlying the associations between DFI and CKD in hypertensive and nonhypertensive individuals are unclear, and the possible reasons are as follows. First, according to previous studies, a higher DFI improves hypertension [12]. Among all participants in this study, the prevalence of hypertension was 40.59% in the lower-DFI group (T1) and 35.65% in the higher-DFI group (T3) (*p* < 0.001), suggesting a negative correlation between DFI and the prevalence of hypertension (Table 2). Since hypertension itself has been identified as a risk factor for the development of CKD, the difference in the prevalence of DFI and CKD between the hypertensive and nonhypertensive groups may be due to the alleviating effect of DFI on hypertension. Moreover, our study revealed that those with higher economic incomes had higher dietary fiber intake, which is consistent with the findings of previous studies [32]. This may be because people with higher PIRs are more likely to purchase higher-priced foods, such as vegetables and fruits, and are therefore more inclined to consume dietary fiber, resulting in healthier blood pressure. Second, we aimed to investigate this mechanism by analyzing differences in certain variables between the two participant groups. Through further analysis, we observed that the prevalence of DM, the SII, and BMI were greater in hypertensive individuals than in nonhypertensive individuals (*p* < 0.05) (Table S7). Additionally, DFI was inversely related to the prevalence of DM, the SII, and BMI (Table S8). Previous studies have shown that DM, inflammation, and obesity all contribute to the development of CKD. They are also linked to hypertension and dietary fiber, as detailed below.

First, continuous low-level inflammation plays a significant role in the initiation and progression of CKD, leading to complications such as renal fibrosis and accelerated decline in renal function [33]. Hypertension can trigger a systemic inflammatory response, and interventions that mitigate inflammation can markedly alleviate the effects of hypertension on renal and vascular damage [34]. Some studies have noted lower levels of inflammation in individuals with a high DFI, attributed mainly to a decrease in the production of inflammatory cytokines facilitated by gut microbe-mediated reductions in gut membrane permeability and pH [35–36]. Second, DM is widely recognized as a major risk factor for CKD and albuminuria, and it continues to be the primary cause of kidney failure in the US [37]. Hypertension is closely associated with an increased possibility of developing DM. A large-scale cohort study conducted in the UK suggested that increases in both SBP and DBP were linked to a greater likelihood of developing new-onset type 2 DM [38]. The inclusion of a high-fiber diet as part of DM management is crucial, as individuals transitioning from low to moderate or high intakes can achieve better glycemic control [39]. Third, obesity and CKD are closely linked. Clinically, CKD associated with obesity is characterized by proteinuria, glomerulomegaly, progressive glomerulosclerosis, and reduced kidney function [40]. Obesity is commonly recognized as a risk factor for hypertension, but studies have shown that hypertension itself may in turn lead to obesity. People of the same weight gain more weight in the future if they initially have a higher BP [41]. DFI plays a protective role in preventing obesity, as a high level of fiber intake can diminish the possibility of weight gain or developing obesity by approximately 30% [42].

Taking the above three points into account, DM, inflammation, and obesity are risk factors for CKD. Hypertension can promote the occurrence of these factors, while dietary fiber has an inhibitory effect on them. This may contribute to the difference in the relationship between DFI and CKD in hypertensive and nonhypertensive participants. In addition, the difference may also be related to medical reasons. Calcium channel blockers, as commonly used antihypertensive drugs, block calcium channels in smooth muscle, leading to slower intestinal motility and increasing the duration for which dietary fiber remains in the intestine, thereby amplifying the effect of dietary fiber [43]. This effect is consistent with our findings that an increased DFI is associated with a lower prevalence of CKD only in hypertensive individuals. Based on our findings and relevant guidelines, we recommend that hypertensive patients increase the consumption of fiber-rich foods such as fruits, vegetables, whole grains and legumes, choose antihypertensive medications appropriately and maintain their blood pressure within an appropriate range to reduce the likelihood of developing CKD [44].

In addition, considering the adverse impact of hypertension on CKD, we explored the association between DFI and BP in individuals with hypertension. The findings indicated that while the prevalence of hypertension was dramatically lower among those with a high DFI (T3) than among those with a low DFI (T1) across all participants (Table 2), there were no significant differences in systolic, diastolic or mean arterial pressure between T3 and T1 among hypertensive individuals (Table S9). This result implies that individuals with preexisting hypertension cannot rely solely on increasing their DFI to effectively lower their BP.

In conclusion, our study suggests that an increasing DFI may contribute to a reduction in the occurrence of CKD among individuals with hypertension. According to the recommendations of the American Dietetic Association (ADA), the recommended DFI for men and women is 25 and 38 g, respectively. In our study, we observed that the mean DFI among hypertensive patients (16.57 ± 8.46 g) was significantly lower than the recommended intake. Therefore, it is suggested that the DFI of hypertensive patients should be increased appropriately to reduce the likelihood of CKD.

Our findings suggest that increased DFI intake in hypertensive individuals may reduce the odds of developing CKD, and this result may inform nutritional intervention strategies for hypertensive patients to reduce the prevalence of CKD and associated health care costs. Our study is the first to use the NHANES database to analyze the relationship between DFI and CKD prevalence, providing insight into the role of dietary fiber in kidney disease. In previous studies, the relationship between DFI and CKD has been explored; however, these studies predominantly considered general populations without distinguishing between hypertensive and nonhypertensive subgroups. Our study uniquely contributes to the literature by specifically examining these subgroups, revealing differential impacts of DFI on CKD prevalence. However, there are certain limitations that should be acknowledged. First, as a cross-sectional study, we can establish only an association between DFI and CKD in hypertensive patients rather than a cause–effect relationship. Second, despite our efforts to adjust for some pertinent confounders, it is possible that there are additional unmeasured confounders that could impact the conclusions. Third, dietary fiber data were collected using the average of two 24-h dietary recall surveys, which may cause an inaccurate estimation of DFI. Finally, the exclusion of 9278 individuals due to absent or inaccessible data may decrease the generalizability of our results.

## 5. Conclusions

We discovered that DFI was negatively related to the prevalence of CKD in adult hypertensive patients in the US, but this negative association was not present in the nonhypertensive population. As DFI increases, there is a decreasing trend in the proportion of individuals who are at moderate, high or very high risk of CKD progression. Our findings provide new insights into prevention strategies for CKD.

## Supplementary Material

Table S7 The DM prevalence SII and BMI by hypertension and nonhypertension weighted.xlsx

Figure S2 Restricted cubic spline plot of the association between DFI and UACR.jpg

Table S2 Fundamental attributes of weighted samples without hypertension from the CKD and non_CKD groups.xlsx

Table S6 Correlations between DFI and UACR weighted.xlsx

Table S3 Fundamental attributes of the weighted sample with hypertension by DFI tertiles.xlsx

Table S1 Fundamental attributes of weighted samples with hypertension from the CKD and non_CKD groups.xlsx

Table S8 Correlations between DFI tertiles and DM SII and BMI weighted.xlsx

Supplementary Material revised second revision.docx

Figure S1 Restricted cubic spline plot of the association between DFI and eGFR.jpg

Table S5 Correlations between DFI and eGFR weighted.xlsx

Table S4 Fundamental attributes of the weighted sample without hypertension by DFI tertiles.xlsx

Table S9 The blood pressure of the weighted sample with hypertension by DFI tertiles.xlsx
