# Supplementary figures and images for: Association between dietary fiber intake and chronic kidney disease in adults with and without hypertension in the United States: a cross-sectional study of NHANES 2009–2020
Source: Ren Fail. 2024 Oct 16;46(2):2415514. doi: 10.1080/0886022X.2024.2415514 (PMC11486002; doi:10.1080/0886022X.2024.2415514)

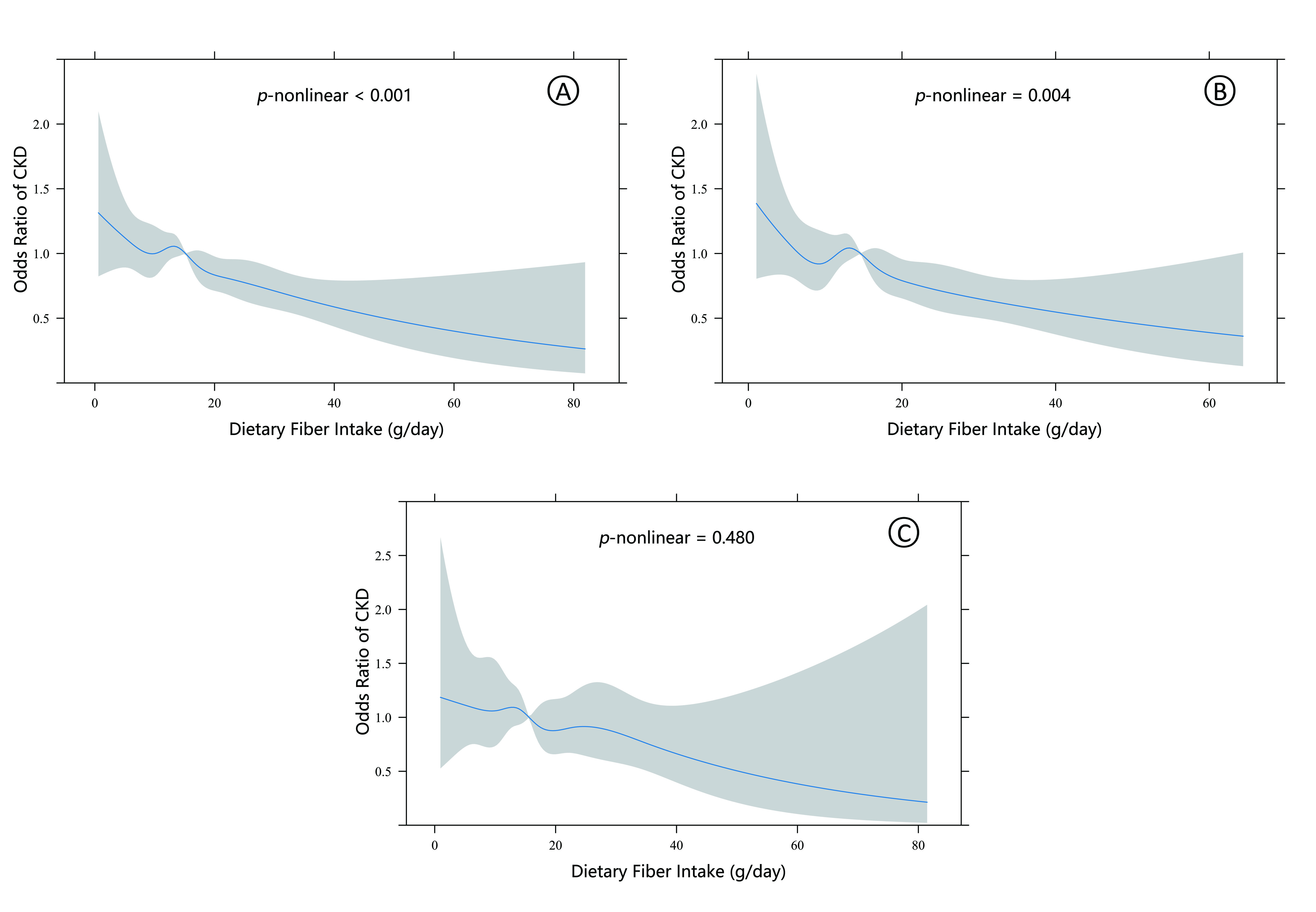

Supplement: Figure S2 Restricted cubic spline plot of the association between DFI and UACR.jpg [file IRNF_A_2415514_SM4702.jpg]

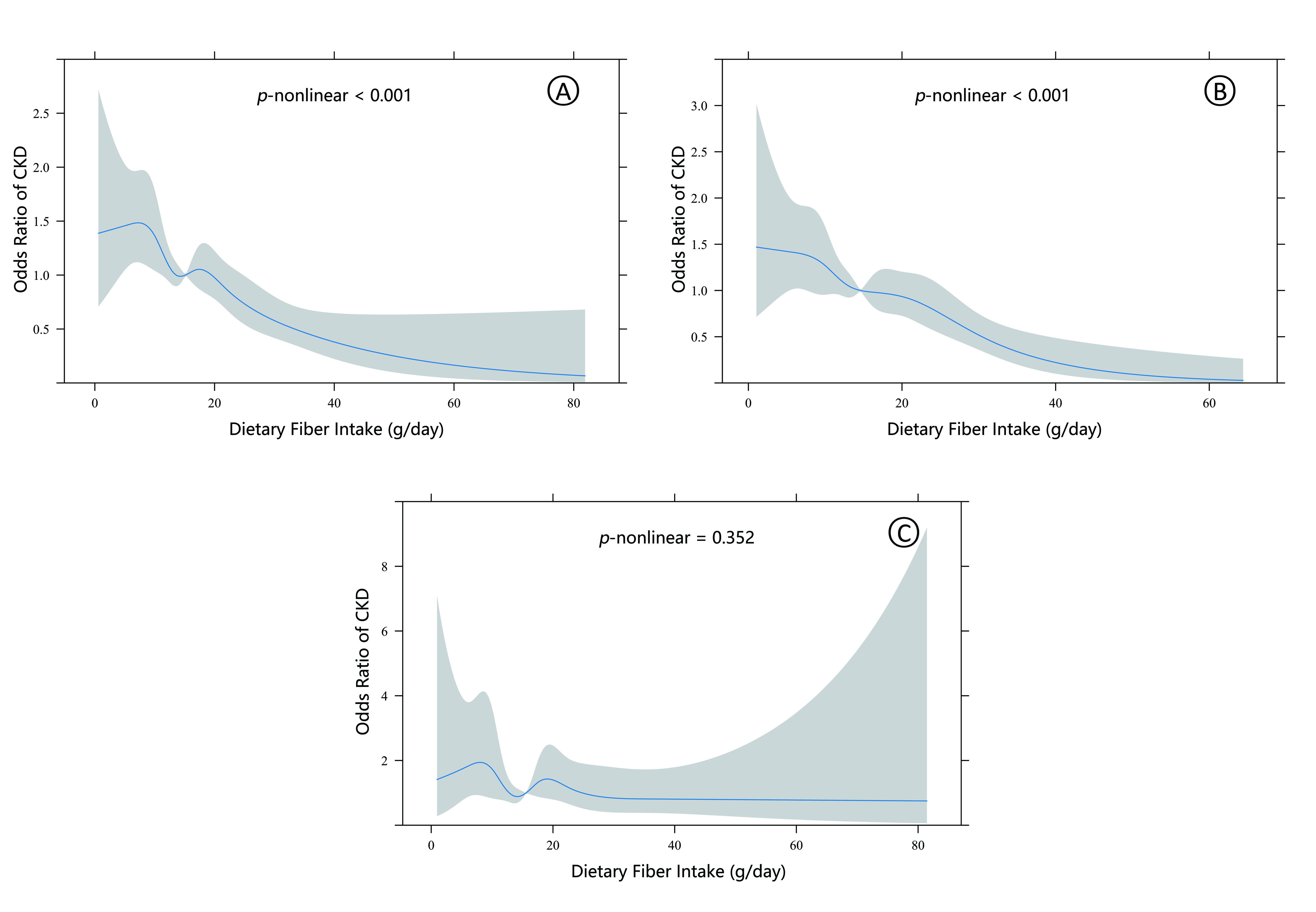

Supplement: Figure S1 Restricted cubic spline plot of the association between DFI and eGFR.jpg [file IRNF_A_2415514_SM4695.jpg]
