# Supplementary material for: Association between dietary fiber intake and chronic kidney disease in adults with and without hypertension in the United States: a cross-sectional study of NHANES 2009–2020
Source: Ren Fail. 2024 Oct 16;46(2):2415514. doi: 10.1080/0886022X.2024.2415514 (PMC11486002; doi:10.1080/0886022X.2024.2415514)
Supplement: Supplementary Material revised second revision.docx [file IRNF_A_2415514_SM4696.docx]

**Figures**


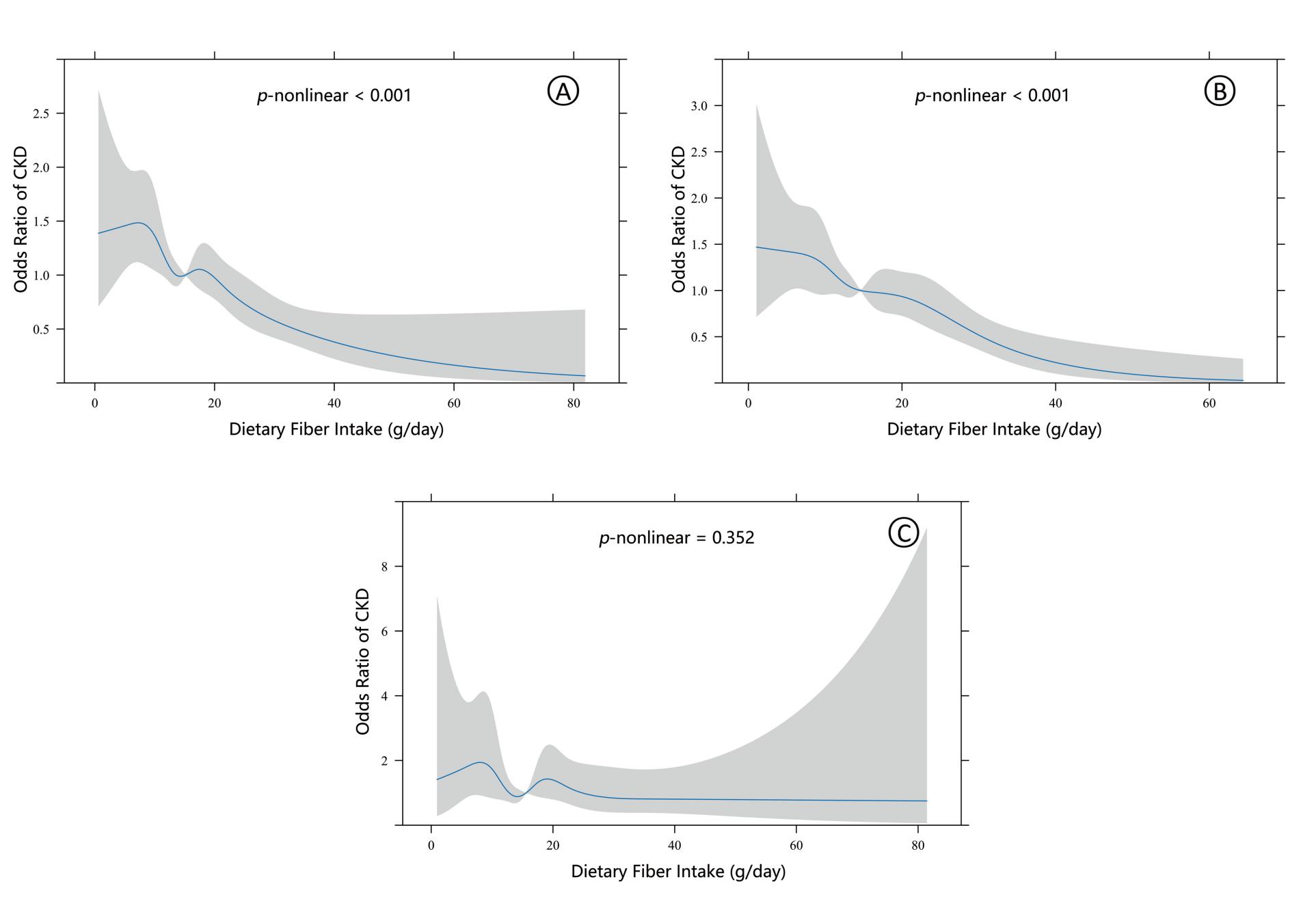


Figure S1. Restricted cubic spline (RCS) plot of the association between DFI and eGFR (< 60 and ≥ 60 ml/min/1.73 m^2^). (A) In all participants, there was a nonlinear inverse association between DFI and eGFR; (B) in patients with hypertension, there was a nonlinear inverse association between DFI and eGFR; (C) in patients without hypertension, there was no non-linear inverse association between DFI and eGFR. The adjustment factors are the same as those presented in Model 3. The solid line and shading represent the odds ratio and its 95% confidence intervals, respectively.


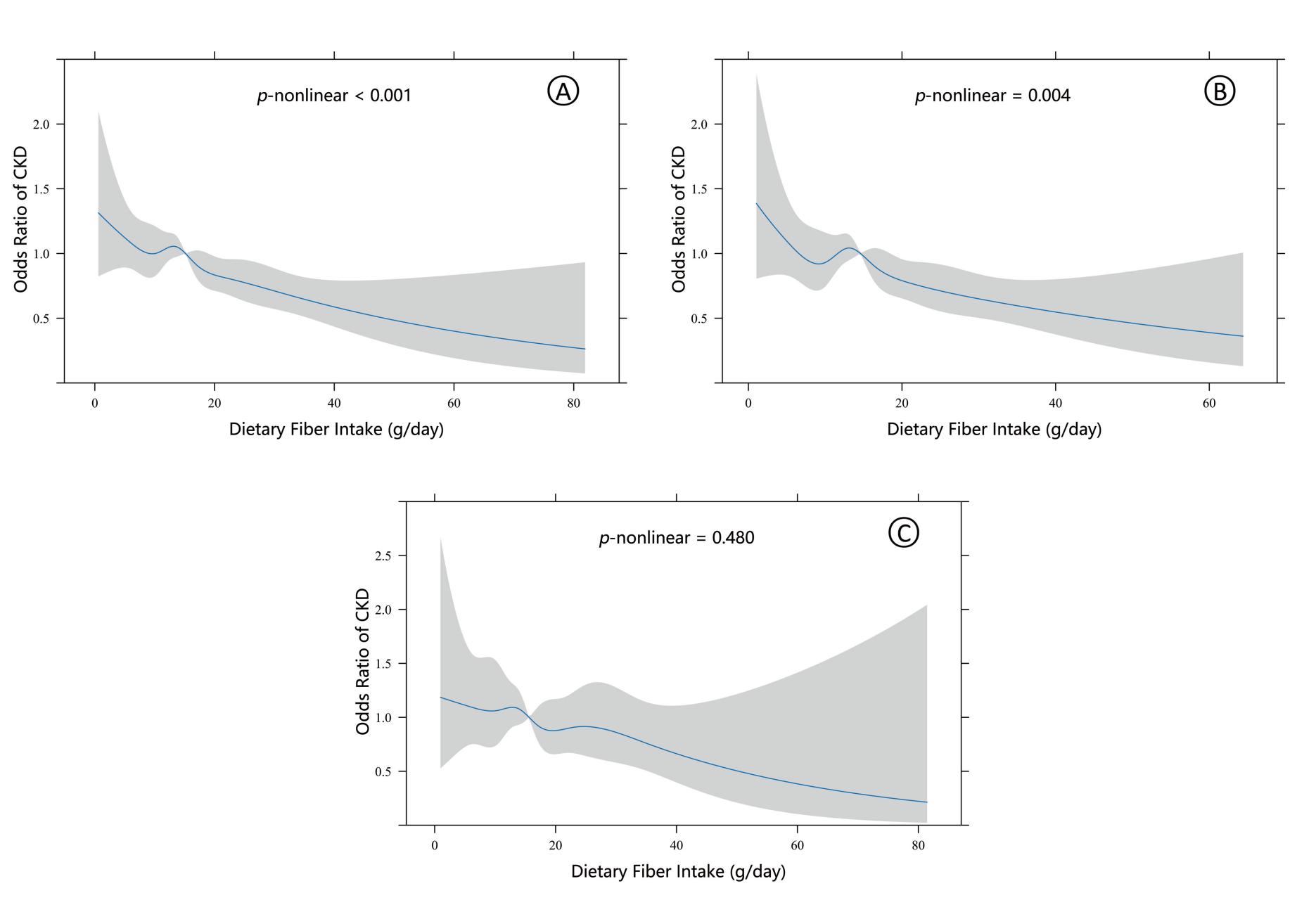


Figure S2. Restricted cubic spline (RCS) plot of the association between DFI and UACR (< 30 and ≥ 30mg/g). (A) In all participants, there was a nonlinear inverse association between DFI and UACR; (B) in patients with hypertension, there was a nonlinear inverse association between DFI and UACR; (C) in patients without hypertension, there was no nonlinear inverse association between DFI and UACR. The adjustment factors are the same as those presented in Model 3. The solid line and shading represent the odds ratio and its 95% confidence intervals, respectively.

**Tables**

|  | Overall | CKD | Non-CKD | *p*-value |
| --- | --- | --- | --- | --- |
|  | N = 9861 | N = 2465 | N = 7396 |  |
| Sex (%) |  |  |  | 0.022 |
| Male | 49.04 | 52.05 | 48.28 |  |
| Female | 50.96 | 47.95 | 51.72 |  |
| Age (years) | 58.06 ± 14.67 | 64.82 ± 13.59 | 56.35 ± 14.43 | < 0.001 |
| Race (%) |  |  |  | < 0.001 |
| Mexican American | 5.52 | 6.26 | 5.33 |  |
| Non-Hispanic Black | 13.29 | 16.49 | 12.48 |  |
| Non Hispanic White | 69.65 | 66 | 70.57 |  |
| Other Hispanic | 4.97 | 4.36 | 5.13 |  |
| Other races | 6.58 | 6.9 | 6.5 |  |
| Education (%) |  |  |  | < 0.001 |
| College or more | 58.76 | 52.98 | 60.22 |  |
| High school | 25.69 | 26.06 | 25.6 |  |
| Middleschool or lower | 15.47 | 20.86 | 14.11 |  |
| Marital status (%) |  |  |  | < 0.001 |
| Never married | 15.42 | 14.05 | 15.77 |  |
| Married or with partner | 71.61 | 65.15 | 73.24 |  |
| Widowed,divorced or separated | 12.97 | 20.81 | 10.99 |  |
| PIR | 3.01 ± 1.62 | 2.71 ± 1.60 | 3.08 ± 1.62 | < 0.001 |
| Smoke status (%) |  |  |  | < 0.001 |
| Never smokers | 50.01 | 45.91 | 51.04 |  |
| Ever smokers | 32.81 | 38.46 | 31.38 |  |
| Current smokers | 17.19 | 15.63 | 17.58 |  |
| Alcohol consumption (%) |  |  |  | 0.196 |
| Mild | 89.17 | 90.99 | 88.71 |  |
| Moderate | 6.14 | 5.02 | 6.42 |  |
| Heavy | 4.69 | 3.99 | 4.87 |  |
| Dietary energy intake (kcal) | 2022.58 ± 782.28 | 1873.00 ± 732.93 | 2060.26 ± 789.82 | < 0.001 |
| DM (%) |  |  |  | < 0.001 |
| Yes | 23.87 | 42.04 | 19.28 |  |
| No | 76.13 | 57.96 | 80.72 |  |
| BMI (%) |  |  |  | 0.077 |
| < 25 | 16.83 | 15.32 | 17.21 |  |
| 25 - 30 | 33.97 | 32.32 | 34.37 |  |
| ≥ 30 | 49.2 | 52.36 | 48.42 |  |
| Sedentary time (%) |  |  |  | 0.005 |
| < 3 hours/day | 9.61 | 8.51 | 9.89 |  |
| 3 - 6 hours/day | 47.38 | 43.9 | 48.25 |  |
| ≥ 6 hours/day | 43.01 | 47.6 | 41.86 |  |
| Hypercholesterolemia (%) |  |  |  | 0.956 |
| Yes | 12.98 | 13.03 | 12.97 |  |
| No | 87.02 | 86.97 | 87.03 |  |
| Cardiac disease (%) |  |  |  | < 0.001 |
| Yes | 5.09 | 11.03 | 3.59 |  |
| No | 94.91 | 88.97 | 96.41 |  |
| Cancer (%) |  |  |  | < 0.001 |
| Yes | 16.74 | 21.69 | 15.5 |  |
| No | 83.26 | 78.31 | 84.5 |  |
| DFI (g/day) | 16.57 ± 8.46 | 15.32 ± 7.67 | 16.89 ± 8.62 | < 0.001 |

Refer to Table 1 for details.

Table S1. Fundamental attributes of weighted samples with hypertension from the CKD and non-CKD groups

|  | Overall | CKD | Non-CKD | *p*-value |
| --- | --- | --- | --- | --- |
|  | N = 13010 | N = 989 | N = 12021 |  |
| Sex (%) |  |  |  | 0.078 |
| Male | 47.53 | 43.98 | 47.77 |  |
| Female | 52.47 | 56.02 | 52.23 |  |
| Age (years) | 42.49 ± 15.25 | 48.17 ± 18.53 | 42.11 ± 14.92 | < 0.001 |
| Race (%) |  |  |  | 0.199 |
| Mexican American | 9.56 | 11.09 | 9.45 |  |
| Non-Hispanic Black | 8.89 | 10.18 | 8.8 |  |
| Non Hispanic White | 66.8 | 64.64 | 66.95 |  |
| Other Hispanic | 6.59 | 6.7 | 6.58 |  |
| Other races | 8.16 | 7.39 | 8.22 |  |
| Education (%) |  |  |  | < 0.001 |
| College or more | 67.22 | 58.47 | 67.82 |  |
| High school | 20.99 | 24.05 | 20.78 |  |
| Middleschool or lower | 11.74 | 17.42 | 11.36 |  |
| Marital status (%) |  |  |  | 0.001 |
| Never married | 13.1 | 13.97 | 13.03 |  |
| Married or with partner | 83.07 | 78.23 | 83.42 |  |
| Widowed,divorced or separated | 3.83 | 7.79 | 3.55 |  |
| PIR | 3.11 ± 1.65 | 2.76 ± 1.67 | 3.14 ± 1.65 | < 0.001 |
| Smoke status (%) |  |  |  | 0.136 |
| Never smokers | 60.55 | 58.7 | 60.67 |  |
| Ever smokers | 21.47 | 24.55 | 21.27 |  |
| Current smokers | 17.98 | 16.75 | 18.06 |  |
| Alcohol consumption (%) |  |  |  | 0.007 |
| Mild | 85.73 | 90.02 | 85.44 |  |
| Moderate | 9.75 | 5.72 | 10.02 |  |
| Heavy | 4.52 | 4.26 | 4.54 |  |
| Dietary energy intake (kcal) | 2129.61 ± 819.8 | 2025.39 ± 827.78 | 2136.67 ± 818.81 | 0.001 |
| DM (%) |  |  |  | < 0.001 |
| Yes | 5.42 | 19.79 | 4.46 |  |
| No | 94.58 | 80.21 | 95.54 |  |
| BMI (%) |  |  |  | 0.001 |
| < 25 | 35.03 | 36.13 | 34.95 |  |
| 25 - 30 | 40.18 | 33.15 | 40.66 |  |
| ≥ 30 | 24.79 | 30.72 | 24.39 |  |
| Sedentary time (%) |  |  |  | 0.378 |
| < 3 hours/day | 12.95 | 13.74 | 12.89 |  |
| 3 - 6 hours/day | 45.82 | 47.86 | 45.68 |  |
| ≥ 6 hours/day | 41.23 | 38.4 | 41.42 |  |
| Hypercholesterolemia (%) |  |  |  | 0.582 |
| Yes | 11.29 | 12.09 | 11.23 |  |
| No | 88.71 | 87.91 | 88.77 |  |
| Cardiac disease (%) |  |  |  | < 0.001 |
| Yes | 0.69 | 4.36 | 0.44 |  |
| No | 99.31 | 95.64 | 99.56 |  |
| Cancer (%) |  |  |  | 0.007 |
| Yes | 7.4 | 10.17 | 7.22 |  |
| No | 92.6 | 89.83 | 92.78 |  |
| DFI (g/day) | 17.68 ± 9.70 | 17.00 ± 9.09 | 17.72 ± 9.74 | 0.097 |

Refer to Table 1 for details.

Table S2. Fundamental attributes of weighted samples without hypertension from the CKD and non-CKD groups

|  | Overall | T1 < 11.55 g/day | T2 11.55~18.08 g/day | T3 ≥ 18.08 g/day | *p*-value |
| --- | --- | --- | --- | --- | --- |
|  | N = 9861 | N = 3240 | N = 3268 | N = 3353 |  |
| Sex (%) |  |  |  |  | < 0.001 |
| Male | 49.04 | 38.15 | 46.8 | 60.23 |  |
| Female | 50.96 | 61.85 | 53.2 | 39.77 |  |
| Age (years) | 58.06 ± 14.67 | 57.29 ± 15.29 | 58.74 ± 14.68 | 58.05 ± 14.09 | 0.005 |
| Race (%) |  |  |  |  | < 0.001 |
| Mexican American | 5.52 | 3.56 | 5.03 | 7.61 |  |
| Non-Hispanic Black | 13.29 | 19.12 | 12.26 | 9.43 |  |
| Non Hispanic White | 69.65 | 66.12 | 71.85 | 70.46 |  |
| Other Hispanic | 4.97 | 5 | 4.55 | 5.36 |  |
| Other races | 6.58 | 6.2 | 6.31 | 7.15 |  |
| Education (%) |  |  |  |  | < 0.001 |
| College or more | 58.76 | 48.52 | 56.92 | 69.04 |  |
| High school | 25.69 | 30.73 | 29.22 | 18.13 |  |
| Middleschool or lower | 15.47 | 20.73 | 13.78 | 12.72 |  |
| Marital status (%) |  |  |  |  | < 0.001 |
| Never married | 15.42 | 18.72 | 16.22 | 12.26 |  |
| Married or with partner | 71.61 | 65.77 | 69.72 | 77.64 |  |
| Widowed,divorced or separated | 12.97 | 15.51 | 14.07 | 10.1 |  |
| PIR | 3.01±1.62 | 2.55±1.59 | 3.02±1.60 | 3.36±1.58 | < 0.001 |
| Smoke status (%) |  |  |  |  | < 0.001 |
| Never smokers | 50.01 | 46.62 | 50.81 | 52.04 |  |
| Ever smokers | 32.81 | 27.86 | 33.47 | 36.28 |  |
| Current smokers | 17.19 | 25.52 | 15.72 | 11.67 |  |
| Alcohol consumption (%) |  |  |  |  | 0.004 |
| Mild | 89.17 | 86.4 | 91.11 | 89.51 |  |
| Moderate | 6.14 | 6.91 | 5.21 | 6.4 |  |
| Heavy | 4.69 | 6.69 | 3.68 | 4.08 |  |
| Dietary energy intake (kcal) | 2022.58 ± 782.28 | 1552.53 ± 599.35 | 1972.76 ± 627.4 | 2460.54 ± 808.10 | < 0.001 |
| DM (%) |  |  |  |  | 0.086 |
| Yes | 23.87 | 25.98 | 23.32 | 22.63 |  |
| No | 76.13 | 74.02 | 76.68 | 77.37 |  |
| BMI (%) |  |  |  |  | 0.266 |
| < 25 | 16.83 | 17.34 | 16.24 | 16.96 |  |
| 25 - 30 | 33.97 | 33.4 | 32.55 | 35.78 |  |
| ≥ 30 | 49.2 | 49.25 | 51.21 | 47.25 |  |
| Sedentary time (%) |  |  |  |  | 0.283 |
| < 3 hours/day | 9.61 | 10.41 | 9.04 | 9.49 |  |
| 3 - 6 hours/day | 47.38 | 47.67 | 48.84 | 45.75 |  |
| ≥ 6 hours/day | 43.01 | 41.92 | 42.13 | 44.76 |  |
| Hypercholesterolemia (%) |  |  |  |  | 0.388 |
| Yes | 12.98 | 13.09 | 13.74 | 12.16 |  |
| No | 87.02 | 86.91 | 86.26 | 87.84 |  |
| Cardiac disease (%) |  |  |  |  | 0.02 |
| Yes | 5.09 | 6.04 | 5.22 | 4.17 |  |
| No | 94.91 | 93.96 | 94.78 | 95.83 |  |
| Cancer (%) |  |  |  |  | 0.486 |
| Yes | 16.74 | 15.94 | 17.53 | 16.66 |  |
| No | 83.26 | 84.06 | 82.47 | 83.34 |  |
| CKD (%) |  |  |  |  | < 0.001 |
| Yes | 20.12 | 23.67 | 20.36 | 16.95 |  |
| No | 79.88 | 76.33 | 79.64 | 83.05 |  |
| Refer to Table 2 for details. |  |  |  |  |  |

Table S3. Fundamental attributes of the weighted sample with hypertension by DFI tertiles

|  | Overall | T1 < 12.35 g/day | T2 12.35~19.35 g/day | T3 ≥ 19.35 g/day | *p*-value |
| --- | --- | --- | --- | --- | --- |
|  | N = 13010 | N = 4262 | N = 4324 | N = 4424 |  |
| Sex (%) |  |  |  |  | < 0.001 |
| Male | 47.53 | 39.05 | 45.34 | 57.39 |  |
| Female | 52.47 | 60.95 | 54.66 | 42.61 |  |
| Age (years) | 42.49 ± 15.25 | 40.64 ± 15.34 | 42.91 ± 15.29 | 43.75 ± 14.96 | < 0.001 |
| Race (%) |  |  |  |  | < 0.001 |
| Mexican American | 9.56 | 6.95 | 8.93 | 12.54 |  |
| Non-Hispanic Black | 8.89 | 13.09 | 8.96 | 5.01 |  |
| Non Hispanic White | 66.8 | 65.23 | 68.32 | 66.72 |  |
| Other Hispanic | 6.59 | 7.13 | 5.93 | 6.75 |  |
| Other races | 8.16 | 7.6 | 7.86 | 8.98 |  |
| Education (%) |  |  |  |  | <0.001 |
| College or more | 67.22 | 59.22 | 67.57 | 74.13 |  |
| High school | 20.99 | 27.08 | 21.69 | 14.78 |  |
| Middleschool or lower | 11.74 | 13.6 | 10.73 | 11.05 |  |
| Marital status (%) |  |  |  |  | < 0.001 |
| Never married | 13.1 | 16.83 | 13.03 | 10.6 |  |
| Married or with partner | 83.07 | 77.99 | 82.93 | 86.68 |  |
| Widowed,divorced or separated | 3.83 | 5.18 | 4.05 | 2.72 |  |
| PIR | 3.11 ± 1.65 | 2.80 ± 1.66 | 3.19 ± 1.61 | 3.31 ± 1.64 | < 0.001 |
| Smoke status (%) |  |  |  |  | < 0.001 |
| Never smokers | 60.55 | 53.77 | 61.49 | 65.74 |  |
| Ever smokers | 21.47 | 20.06 | 21.38 | 22.85 |  |
| Current smokers | 17.98 | 26.17 | 17.12 | 11.41 |  |
| Alcohol consumption (%) |  |  |  |  | < 0.001 |
| Mild | 85.73 | 82.5 | 86.57 | 87.54 |  |
| Moderate | 9.75 | 11.63 | 9.33 | 8.64 |  |
| Heavy | 4.52 | 5.87 | 4.1 | 3.83 |  |
| Dietary energy intake (kcal) | 2129.61 ± 819.80 | 1631.79 ± 574.88 | 2112.60 ± 644.02 | 2597.37 ± 890.48 | < 0.001 |
| DM (%) |  |  |  |  | 0.824 |
| Yes | 5.42 | 5.5 | 5.55 | 5.23 |  |
| No | 94.58 | 94.5 | 94.45 | 94.77 |  |
| BMI (%) |  |  |  |  | < 0.001 |
| < 25 | 35.03 | 33.37 | 32.84 | 38.7 |  |
| 25 - 30 | 40.18 | 39.98 | 40.67 | 39.87 |  |
| ≥ 30 | 24.79 | 26.64 | 26.49 | 21.43 |  |
| Sedentary time (%) |  |  |  |  | 0.03 |
| < 3 hours/day | 12.95 | 14.14 | 12.41 | 12.4 |  |
| 3 - 6 hours/day | 45.82 | 47.17 | 44.61 | 45.8 |  |
| ≥ 6 hours/day | 41.23 | 38.69 | 42.98 | 41.8 |  |
| Hypercholesterolemia (%) |  |  |  |  | 0.798 |
| Yes | 11.29 | 11.2 | 11.64 | 11.02 |  |
| No | 88.71 | 88.8 | 88.36 | 88.98 |  |
| Cardiac disease (%) |  |  |  |  | 0.077 |
| Yes | 0.69 | 0.88 | 0.77 | 0.44 |  |
| No | 99.31 | 99.12 | 99.23 | 99.56 |  |
| Cancer (%) |  |  |  |  | 0.082 |
| Yes | 7.4 | 6.36 | 7.93 | 7.83 |  |
| No | 92.6 | 93.64 | 92.07 | 92.17 |  |
| CKD (%) |  |  |  |  | 0.125 |
| Yes | 6.35 | 7.22 | 5.84 | 6.06 |  |
| No | 93.65 | 92.78 | 94.16 | 93.94 |  |
| Refer to Table 2 for details. |  |  |  |  |  |

Table S4. Fundamental attributes of the weighted sample without hypertension by DFI tertiles

|  | OR (95% CI) | | |
| --- | --- | --- | --- |
| All participants | Model 1 | Model 2 | Model 3 |
| DFI (g/day) | 0.97 (0.96, 0.98) | 0.96 (0.95, 0.98) | 0.96 (0.94, 0.98) |
| *p*-value | < 0.001 | < 0.001 | < 0.001 |
| DFI tertiles |  |  |  |
| T1, < 12.00 g/day | Ref | Ref | Ref |
| T2, 12.00 - 18.75 g/day | 0.78 (0.64, 0.94) | 0.74 (0.56, 0.97) | 0.74 (0.54, 1.02) |
| T3, ≥ 18.75 g/day | 0.56 (0.45, 0.70) | 0.51 (0.39, 0.68) | 0.54 (0.39, 0.74) |
| *p* for trend | < 0.001 | < 0.001 | < 0.001 |
|  |  |  |  |
| Hypertensive participants |  |  |  |
| DFI (g/day) | 0.96 (0.96, 0.98) | 0.96 (0.94, 0.97) | 0.96 (0.94, 0.97) |
| *p*-value | < 0.001 | < 0.001 | < 0.001 |
| DFI tertiles |  |  |  |
| T1, < 11.55 g/day | Ref | Ref | Ref |
| T2, 11.55 - 18.08 g/day | 0.76 (0.61, 0.94) | 0.79 (0.61, 1.03) | 0.82 (0.61, 1.09) |
| T3, ≥ 18.08 g/day | 0.57 (0.45, 0.71) | 0.51 (0.38, 0.67) | 0.53 (0.38, 0.75) |
| *p* for trend | < 0.001 | < 0.001 | < 0.001 |
|  |  |  |  |
| Nonhypertensive participants |  |  |  |
| DFI (g/day) | 0.99 (0.97, 1.01) | 0.98(0.95,1.01) | 0.97(0.94,1.01) |
| *p*-value | 0.17 | 0.179 | 0.132 |
| DFI tertiles |  |  |  |
| T1, <12.35 g/day | Ref | Ref | Ref |
| T2, 12.35 - 19.35 g/day | 1.05 (0.65, 1.70) | 0.74 (0.37, 1.63) | 0.78 (0.36, 1.73) |
| T3, ≥ 19.35 g/day | 0.81 (0.50, 1.29) | 0.53 (0.26, 1.14) | 0.52 (0.22, 1.21) |
| *p* for trend | 0.372 | 0.11 | 0.127 |
| Refer to Table 3 for details. |  |  |  |

Table S5. Correlations between DFI and eGFR (< 60 and ≥ 60 ml/min/1.73 m^2^), weighted

|  | OR (95% CI) | | |
| --- | --- | --- | --- |
| All participants | Model 1 | Model 2 | Model 3 |
| DFI (g/day) | 0.98 (0.97, 0.99) | 0.98 (0.97, 0.99) | 0.99 (0.98, 1.00) |
| *p*-value | < 0.001 | 0.002 | 0.046 |
| DFI tertiles |  |  |  |
| T1, < 12.00 g/day | Ref | Ref | Ref |
| T2, 12.00 - 18.75 g/day | 0.78 (0.68, 0.90) | 0.82 (0.69, 0.97) | 0.86 (0.70, 1.06) |
| T3, ≥ 18.75 g/day | 0.66 (0.57, 0.76) | 0.68 (0.55, 0.84) | 0.76 (0.61, 0.96) |
| *p* for trend | < 0.001 | < 0.001 | 0.026 |
|  |  |  |  |
| Hypertensive participants |  |  |  |
| DFI (g/day) | 0.98 (0.97, 0.99) | 0.98 (0.96, 0.99) | 0.98 (0.97, 0.99) |
| *p*-value | < 0.001 | < 0.001 | 0.011 |
| DFI tertiles |  |  |  |
| T1, < 11.55 g/day | Ref | Ref | Ref |
| T2, 11.55 - 18.08 g/day | 0.84 (0.71, 0.99) | 0.82 (0.64, 1.05) | 0.86 (0.64, 1.14) |
| T3, ≥ 18.08 g/day | 0.68 (0.59, 0.78) | 0.68 (0.53, 0.88) | 0.75 (0.58, 0.98) |
| *p* for trend | < 0.001 | 0.005 | 0.042 |
|  |  |  |  |
| Nonhypertensive participants |  |  |  |
| DFI (g/day) | 0.99 (0.98, 1.00) | 0.99 (0.98, 1.01) | 1.00 (0.98, 1.01) |
| *p*-value | 0.17 | 0.335 | 0.659 |
| DFI tertiles |  |  |  |
| T1, <12.35 g/day | Ref | Ref | Ref |
| T2, 12.35 - 19.35 g/day | 0.76 (0.59, 0.98) | 0.82 (0.57, 1.20) | 0.89 (0.59, 1.34) |
| T3, ≥ 19.35 g/day | 0.79 (0.62, 1.02) | 0.84 (0.59, 1.18) | 0.89 (0.60, 1.31) |
| *p* for trend | 0.077 | 0.337 | 0.565 |
| Refer to Table 3 for details. | | | |

Table S6. Correlations between DFI and UACR (< 30 and ≥ 30mg/g), weighted

|  | Overall | Hypertension | Nonhypertension | *p*-value |
| --- | --- | --- | --- | --- |
| DM (%) |  |  |  | < 0.001 |
| Yes | 12.37 | 23.87 | 5.42 |  |
| No | 87.63 | 76.13 | 94.58 |  |
| SII | 525.91 ± 331.40 | 559.19 ± 383.22 | 505.45 ± 293.22 | < 0.001 |
| BMI | 29.34 ± 6.95 | 31.32 ± 7.22 | 28.13 ± 6.49 | < 0.001 |
| DM = diabetes mellitus, SII = systemic immune-inflammation index, BMI = body mass index | | | | |

Table S7. The DM prevalence, SII, and BMI by hypertension and nonhypertension, weighted

|  | OR (95% CI) | | |
| --- | --- | --- | --- |
|  | DM (Yes) | BMI ≥ 30 | SII ≥ 447 |
| DFI tertiles |  |  |  |
| T1, < 12.00 | Ref | Ref | Ref |
| T2, 12.00~18.75 | 0.89 (0.78, 1.01) | 0.90 (0.82, 0.97) | 0.91 (0.83, 1.00) |
| T3, ≥ 18.75 | 0.84 (0.75, 0.94) | 0.69 (0.62, 0.75) | 0.76 (0.69, 0.83) |
| *p* for trend | 0.006 | < 0.001 | < 0.001 |
| DM = diabetes mellitus (Yes, No), BMI = body mass index (< 30, ≥ 30), SII = systemic immune-inflammation index (< 447, ≥ 447) | | | |

Table S8. Correlations between DFI tertiles and DM, SII, and BMI, weighted

|  | Overall | T1 < 11.55 g/day | T2 11.55~18.08 g/day | T3 ≥ 18.08 g/day | *p*-value |
| --- | --- | --- | --- | --- | --- |
|  | N=9861 | N=3240 | N=3268 | N=3353 |  |
| Systolic pressure | 132.53 ± 18.56 | 132.74 ± 19.63 | 132.37 ± 18.38 | 132.50 ± 17.83 | 0.825 |
| Diastolic pressure | 74.00 ± 13.93 | 73.68 ± 14.23 | 74.05 ± 13.89 | 74.22 ± 13.73 | 0.552 |
| Mean arterial pressure | 93.51 ± 12.82 | 93.37 ± 13.24 | 93.49± 12.74 | 93.64 ± 12.54 | 0.864 |

Table S9. The blood pressure of the weighted sample with hypertension by DFI tertiles
